# Individualized Prognostic Insights: CONUT‐GBRS for Survival Prediction in Gallbladder Cancer

**DOI:** 10.1002/cam4.71203

**Published:** 2025-09-12

**Authors:** Si‐qi Yang, Rui‐qi Zou, Yu‐shi Dai, Jun‐ke Wang, Wen‐jie Ma, Hai‐jie Hu, Fu‐yu Li

**Affiliations:** ^1^ Division of Biliary Tract Surgery, Department of General Surgery, West China Hospital Sichuan University Chengdu Sichuan Province China

**Keywords:** controlling nutritional status (CONUT) score, gallbladder cancer, gallbladder cancer predictive risk score (GBRS), prognosis

## Abstract

**Background:**

The most suitable prognostic prediction system for gallbladder cancer (GBC) is yet to be determined. This study aims to establish a combined score integrating preoperative patients' nutritional and immune status and pathological parameters to forecast the survival outcomes following curative‐intent surgery of GBC.

**Methods:**

This retrospective study included patients diagnosed with GBC based on postoperative pathological examinations. The patients underwent curative surgery at West China Hospital of Sichuan University (China) between January 2014 and December 2022. Using the controlling nutritional status (CONUT) score and gallbladder cancer predictive risk score (GBRS), we generated the CONUT‐GBRS for every patient, and the patients were divided into two groups based on the optimal cutoff value. Comparisons were made between the two groups regarding clinicopathologic features and survival.

**Results:**

The optimal cutoff value for the CONUT‐GBRS was 1.39. There were 99 and 201 individuals in the high and low CONUT‐GBRS groups, respectively. Patients with high CONUT‐GBRS experienced poorer overall survival and disease‐free survival compared with those with low CONUT‐GBRS, even after propensity score matching analysis. Both univariate and multivariate Cox analyses established that CONUT‐GBRS stood as an independent prognostic factor for GBC patients. Subgroup analysis indicated that CONUT‐GBRS was also an effective predictor of prognosis in patients with incidental GBC.

**Conclusion:**

The CONUT‐GBRS serves as an advantageous, straightforward, and cost‐effective prognostic tool for GBC, offering valuable prognostic insights and guiding the tailoring of individualized treatment strategies to improve patient outcomes.

## 1 Introduction

Gallbladder carcinoma (GBC) stands as one of the most aggressive malignant tumors, comprising two‐thirds of cancers within the biliary system. While GBC is relatively uncommon, its incidence is on the rise each year, constituting approximately 0.6% of newly diagnosed cancers and 0.9% of cancer‐related deaths worldwide [1]. Due to its distinctive anatomical location and biological features, early detection of GBC is relatively infrequent. Typically, it is serendipitously discovered during or after surgery, and the majority of diagnoses occur in advanced stages, leading to less favorable treatment outcomes [2]. In recent years, there has been remarkable rapid advancement in surgical techniques, and radical surgical treatment has emerged as the most effective approach for GBC. However, the notable challenge lies in the high recurrence rate following surgery, significantly affecting the long‐term survival of patients with GBC [3, 4]. Moreover, chemotherapy, targeted therapy, and immunotherapy have been employed in the treatment of GBC. However, there remains a scarcity of robust evidence‐based medical data supporting the efficacy of these treatments in enhancing patient prognosis.

Numerous studies have explored prognostic factors in GBC, with the American Joint Committee on Cancer (AJCC) TNM staging system being extensively applied in clinical practice. However, the TNM staging system relies solely on dependable pathological parameters and does not encompass tumor heterogeneity and the host's intrinsic factors, restricting its prognostic significance in tumors [5, 6]. Several other pathological factors, such as tumor necrosis, perineural invasion, lymphovascular invasion, and tumor differentiation, have also demonstrated associations with the prognosis of GBC [7, 8, 9]. The gallbladder cancer predictive risk score (GBRS), as introduced by Ethun et al. includes tumor grade, tumor differentiation, perineural invasion, and lymphovascular invasion [10], which has a more significant impact on the prognosis of GBC when compared to the TNM classification [5, 10]. In addition, the patient's intrinsic nutritional and immune status has been demonstrated to impact prognosis. Tumor‐related nutritional and immune biomarkers, such as neutrophil to lymphocyte ratio (NLR), platelet to lymphocyte ratio (PLR), and prognostic nutritional index (PNI), have been employed to predict survival in patients with GBC [11, 12, 13]. Controlling nutritional status (CONUT) score is a recently introduced scoring system that considers both the inflammatory and nutritional status of the patient. It involves assessing serum albumin levels, total cholesterol levels, and total lymphocyte counts in the peripheral blood. Several studies have affirmed its effectiveness as an independent prognostic marker in individuals with tumors [14, 15, 16].

In this study, we investigated the prognostic significance of preoperative CONUT score and GBRS in GBC patients. Additionally, we developed a predictive model that integrates patients' nutritional and immune status along with clinicopathological factors to provide more precise information for prognostic risk stratification and guide personalized treatment approaches.

## 2 Materials and Methods

### 2.1 Study Population

This study retrospectively recruited patients diagnosed with GBC through postoperative pathological examinations who received curative surgery at West China Hospital of Sichuan University (China) from January 2014 to December 2022. The demographic details, laboratory test results, surgical information, and pathological information of all included patients were extracted from the medical record. Patients who either (1) lacked sufficient clinical and follow‐up data, (2) failed to achieve R0 resection, or (3) presented with other tumors were excluded. Approval for this study was granted by the Institutional Ethics Review Board of West China Hospital (20231394), and the requirement for informed consent was waived because this study was retrospective.

### 2.2 Follow‐Up Assessments

Follow‐up was performed from the date of surgery until December 2023. All patients underwent rigorous retrospective follow‐up through outpatient check‐ups or telephone interviews. Patients underwent follow‐up evaluations every 3 months during the first postoperative year, followed by assessments every 6 months thereafter. The follow‐up included physical examinations, liver function, serum level of CA19‐9 and CEA, and abdominal computed tomography (CT) or magnetic resonance imaging (MRI). Overall survival (OS) was characterized as the duration from the surgery date to either death or the last follow‐up, and disease‐free survival (DFS) was defined as the duration from the surgery date to the first recurrence.

### 2.3 Surgical Procedure

All included patients underwent curative surgery, which comprised a minimum of cholecystectomy and lymph node dissection. Supplementary procedures such as hepatectomy, common bile duct resection, and hepaticopancreaticoduodenectomy were determined by surgeons based on the extent of the tumor. The extent of liver resection included wedge resection, SIVB + V resection, right hemi‐hepatectomy, or more extended right hemi‐hepatectomy.

### 2.4 Data Collection

Clinicopathological variables were retrospectively collected, including age, sex, body mass index (BMI), preoperative lymphocyte count, preoperative levels of serum CA19‐9, CA125, CEA, cholesterol, and albumin, gallstones, incidental gallbladder carcinoma (ICGB), liver resection, bile duct resection, postoperative complications, pathology subtype, differentiation, perineural invasion, lymphovascular invasion, lymph node metastasis, and AJCC T‐stage. Postoperative complications were categorized using the Clavien‐Dindo classification. The CONUT score was determined based on the serum albumin level, total lymphocyte count, and cholesterol level [17], and GBRS was calculated with tumor grade, tumor differentiation, perineural invasion, and lymphovascular invasion [10]. The computing formula of CONUT and GBRS was exhibited in Tables S1 and S2.

### 2.5 Statistical Analysis

IBM SPSS 23.0 (Chicago, IL, USA), GraphPad Prism 8, and R statistics software (v4.2.1) were employed for statistical analysis. Continuous and categorical data were exhibited as median (range) and number (percentage), respectively. Group comparisons were performed using Fisher's exact test, chi‐squared test, or Mann–Whitney *U* test, as appropriate. The Kaplan–Meier method was utilized to generate survival curves and was compared using the log‐rank test across various groups. The independent predictors of survival were evaluated by univariate and multivariate Cox regression analyses. A risk score was assigned to each patient based on the formula: CONUT‐GBRS = CONUT * β1 + GBRS * β2 (β1 and β2 were the regression coefficient derived from the Cox regression model). The optimal cutoff value for the risk score was determined through X‐tile software (v3.6.1). Propensity score matching (PSM) was performed to reduce the impact of confounding bias. Statistical significance was determined at a two‐tailed *p*‐value < 0.05.

## 3 Results

### 3.1 Basic Characteristics

This study recruited a total of 300 patients who had undergone radical surgery and achieved R0 resection in our hospital. Among these patients, 121 (40.3%) were male, and 179 (59.7%) were female, with a median age of 62 years (range from 29 to 88) (Table 1). A total of 198 (66.0%) individuals underwent liver resection, and 145 received bile duct resection. Out of 300 patients, 77 (25.6%) encountered postoperative complications (Clavien‐Dindo grade ≥ II). The median OS and DFS of our cohort were 22 and 15 months, respectively.

**TABLE 1:** Baseline characteristics for all included patients.

Variables	All (*n* = 300)	High CONUT‐GBRS (*n* = 99, 33.0%)	Low CONUT‐GBRS (*n* = 201, 67.0%)	*p*
Age (years)				
≤ 60	129 (43.0%)	36 (36.4%)	93 (46.3%)	0.109
> 60	171 (57.0%)	63 (63.6%)	108 (53.7%)	
Sex				
Male	121 (40.3%)	37 (37.4%)	84 (51.7%)	0.532
Female	179 (59.7%)	62 (62.6%)	117 (73.3%)	
BMI (kg/m^2^)				
≤ 23	144 (48.0%)	47 (47.5%)	97 (48.3%)	0.903
> 23	156 (52.0%)	52 (52.5%)	104 (51.7%)	
CEA (ng/mL)				
≤ 5	230 (76.7%)	68 (68.7%)	162 (80.6%)	0.029
> 5	70 (23.3%)	31 (31.3%)	39 (19.4%)	
CA125 (U/mL)				
≤ 24	193 (64.3%)	62 (62.6%)	131 (65.2%)	0.701
> 24	107 (35.7%)	37 (37.4%)	70 (34.8%)	
CA19‐9 (U/mL)				
≤ 30	149 (41.3%)	39 (39.4%)	110 (54.7%)	0.014
> 30	151 (58.7%)	60 (60.6%)	91 (45.3%)	
Gallbladder stones				
Present	145 (48.3%)	46 (46.5%)	99 (49.3%)	0.901
Absent	155 (51.7%)	50 (53.5%)	102 (50.7%)	
Liver resection				
Yes	198 (66.0%)	70 (70.7%)	128 (63.4%)	0.245
No	102 (34.0%)	29 (29.3%)	73 (36.6%)	
Bile duct resection				
Yes	145 (48.3%)	50 (50.5%)	95 (47.3%)	0.624
No	155 (51.7%)	49 (49.5%)	106 (52.7%)	
ICGB				
Yes	103 (34.3%)	21 (21.2%)	82 (40.8%)	0.001
No	197 (65.7%)	78 (78.8%)	119 (59.2%)	
Postoperative complication				
Present	77 (25.6%)	32 (32.3%)	45 (22.4%)	0.069
Absent	223 (74.3%)	67 (67.7%)	156 (77.6%)	
Pathology				
Adenocarcinoma	267 (89.0%)	83 (83.8%)	184 (91.1%)	0.051
Others	33 (11.0%)	16 (16.2%)	17 (8.5%)	
Differentiation				
Poor	139 (46.3%)	61 (61.6%)	78 (38.8%)	0.001
Moderate/well	161 (53.7%)	38 (39.4%)	123 (61.2%)	
Perineural invasion				
Present	49 (16.3%)	29 (29.3%)	20 (10.0%)	0.001
Absent	251 (83.7%)	70 (71.7%)	181 (90.0%)	
Lymphovascular invasion				
Present	54 (18.0%)	19 (19.2%)	35 (17.4%)	0.750
Absent	246 (82.0%)	80 (80.9%)	166 (82.6%)	
Node metastasis				
Persent	117 (39.0%)	46 (46.5%)	71 (35.3%)	0.078
Absent	183 (61.0%)	53 (53.5%)	130 (64.7%)	
pT (8th AJCC)				
T1/T2	228 (76.0%)	67 (67.7%)	161 (80.1%)	0.022
T3	72 (24.0%)	32 (32.3%)	40 (19.9%)	

Abbreviations: AJCC, American Joint Committee on Cancer; BMI, body mass index; CA125, carbohydrate antigen 125; CA19‐9, carbohydrate antigen 19‐9; CEA, carcinoembryonic antigen; ICGB, incidental gallbladder carcinoma.

### 3.2 Construction and Cutoff Value of COUNT‐GBRS

We employed Cox regression analysis to investigate the prognostic value of CONUT and GBRS. The univariate analysis indicated that both CONUT and GBRS were significantly associated with OS and DFS (Table 2). The multivariate analysis suggested that both CONUT and GBRS served as independent prognostic indicators for OS and DFS in our cohort. The CONUT‐GBRS score of every patient was calculated with the formula: CONUT‐GBRS = CONUT * 0.22 + GBRS * 0.16. Based on the ROC curve analysis, the AUC value for CONUT‐GBRS was determined to be 0.758 (Figure S1). The optimal cutoff value for the CONUT‐GBRS was 1.39, calculated by X‐tile software.

**TABLE 2:** Univariate and multivariate analyses of overall survival and disease‐free survival.

Variables	Univariate analysis			Multivariate analysis		
	HR	95% CI	*p*	HR	95% CI	*p*
Overall survival						
Age (> 60 vs. ≤ 60)	1.271	0.948–1.705	0.108	—	—	—
Sex (female vs. male)	1.180	0.882–1.578	0.264	—	—	—
BMI (> 23 vs. ≤ 23)	1.021	0.768–1.359	0.886	—	—	—
CEA (> 5 vs. ≤ 5)	1.839	1.341–2.520	< 0.001	1.893	1.370–2.616	< 0.001
CA125 (> 24 vs. ≤ 24)	1.329	0.994–1.778	0.55	—	—	—
CA19‐9 (> 30 vs. ≤ 30)	1.296	0.974–1.724	0.76	—	—	—
CONUT (> 4 vs. ≤ 4)	1.714	1.286–2.284	< 0.001	1.329	1.242–1.422	< 0.001
Gallbladder stones	1.116	0.839–1.485	0.45	—	—	—
Liver resection	1.076	0.791–1.463	0.64	—	—	—
Bile duct resection	1.14	0.858–1.515	0.367	—	—	—
Postoperative complication	1.194	0.861–1.654	0.288	—	—	—
Pathology (adenocarcinoma vs. other)	1.050	0.653–1.689	0.839	—	—	—
Differentiation (poor vs. moderate/well)	1.219	0.918–1.620	0.172	—	—	—
Perineural invasion (positive vs. negative)	1.897	1.379–2.609	< 0.001	1.162	0.800–1.588	0.430
Lymphovascular invasion (positive vs. negative)	1.102	0.763–1.592	0.604	—	—	—
Node metastasis (positive vs. negative)	1.751	1.312–2.337	< 0.001	1.499	1.105–2.032	0.009
pT (8th AJCC) (T3 vs. T1/T2)	1.656	1.294–2.119	< 0.001	1.964	1.501–2.571	< 0.001
GBRS (> 5 vs. ≤ 5)	2.346	1.698–3.241	< 0.001	2.260	1.523–3.354	< 0.001
Disease‐free survival						
Age (> 60 vs. ≤ 60)	1.526	1.164–2.001	0.002	1.472	1.109–1.955	0.008
Sex (female vs. male)	1.174	0.889–1.551	0.257	—	—	—
BMI (> 23 vs. ≤ 23)	1.185	0.903–1.554	0.221	—	—	—
CEA (> 5 vs. ≤ 5)	1.358	0.999–1.875	0.51	—	—	—
CA125 (> 24 vs. ≤ 24)	1.115	0.842–1.476	0.447	—	—	—
CA19‐9 (> 30 vs. ≤ 30)	1.042	0.796–1.364	0.765	—	—	—
CONUT (> 4 vs. ≤ 4)	1.663	1.273–2.173	< 0.001	1.634	1.224–2.182	0.001
Gallbladder stones	1.039	0.793–1.360	0.783	—	—	—
Liver resection	1.194	0.979–1.456	0.079	—	—	—
Bile duct resection	1.333	1.017–1.746	0.037	1.165	0.884–1.535	0.277
Postoperative complication	1.244	0.917–1.687	0.16	—	—	—
Pathology (adenocarcinoma vs. other)	1.229	0.815–1.855	0.326	—	—	—
Differentiation (poor vs. moderate/well)	1.418	1.082–1.858	0.011	1.327	0.971–1.815	0.076
Perineural invasion (positive vs. negative)	1.642	1.196–2.253	0.002	1.126	0.791–1.603	0.509
Lymphovascular invasion (positive vs. negative)	1.070	0.750–1.526	0.710	—	—	—
Node metastasis (positive vs. negative)	2.436	1.853–3.202	< 0.001	2.351	1.763–3.137	< 0.001
pT (8th AJCC) (T3 vs. T1/T2)	2.306	1.712–3.106	< 0.001	—	—	—
GBRS (> 5 vs. ≤ 5)	2.306	1.712–3.106	< 0.001	2.713	1.881–3.914	< 0.001

Abbreviations: AJCC, American Joint Committee on Cancer; BMI, body mass index; CA125, carbohydrate antigen 125; CA19‐9, carbohydrate antigen 19‐9; CEA, carcinoembryonic antigen; CONUT, controlling nutritional status; GBRS, gallbladder cancer predictive risk score.

### 3.3 Correlations Between the CONUT‐GBRS and Clinicopathological Characteristics

Based on the optimal cutoff value of CONUT‐GBRS, all included GBC patients were stratified into low and high CONUT‐GBRS groups. The clinicopathological features of all included patients, categorized by the CONUT‐GBRS score group, are presented in Table 1. In the high CONUT‐GBRS group, individuals presented with higher serum levels of CEA (*p* = 0.029) and CA19‐9 (*p* = 0.014). In terms of pathological features, high CONUT‐GBRS was correlated with aggressive tumor characteristics, including poor differentiation (*p* = 0.001), presence of perineural invasion (*p* = 0.001) and tumor stage (*p* = 0.038).

### 3.4 Survival Analysis

**FIGURE 1:**
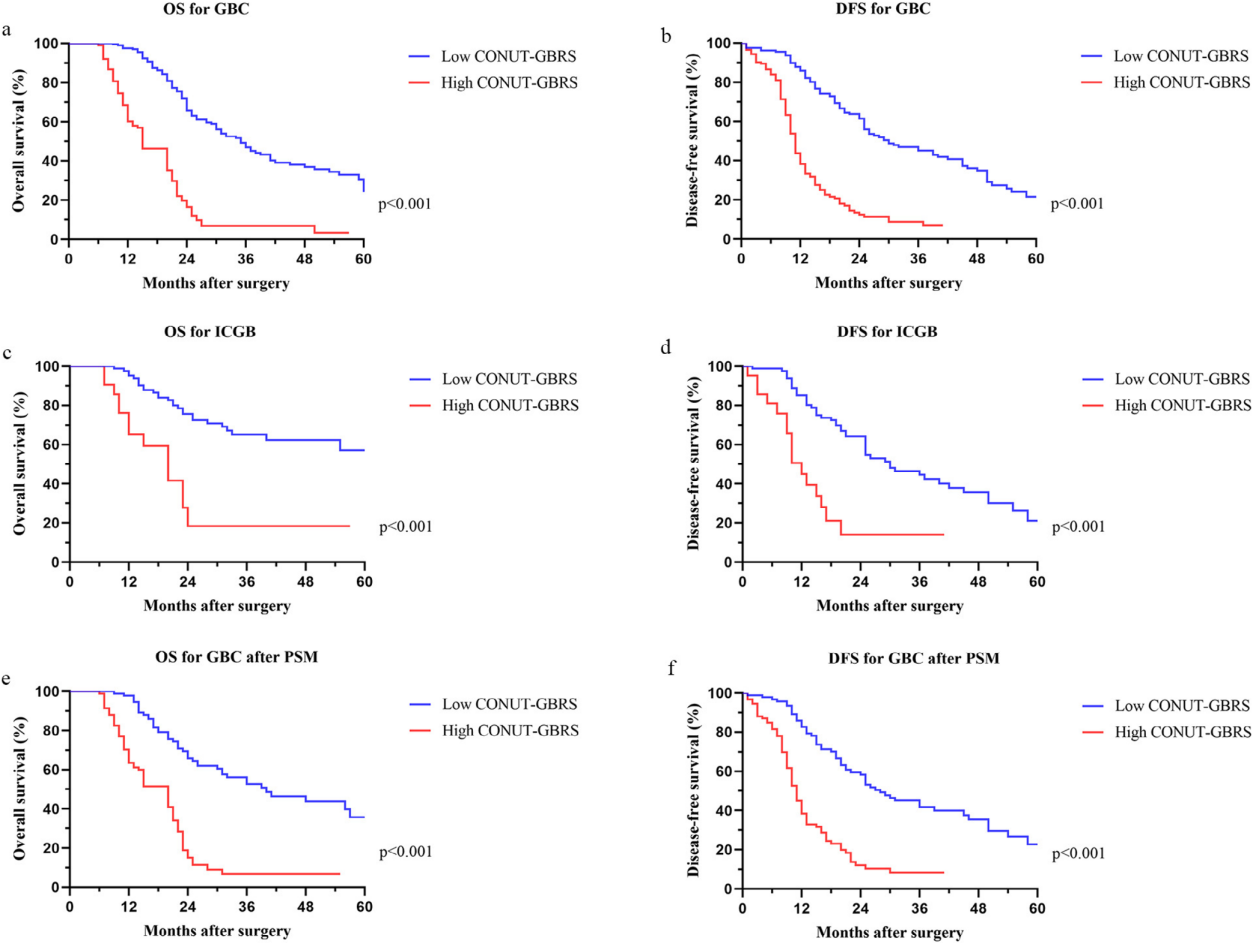
Comparison of survival outcomes between low and high CONUT‐GBRS groups. (a) Overall survival of GBC before PSM. (b) Disease‐free survival of GBC before PSM. (c) Overall survival of IGBC. (d) Disease‐free survival of ICGB. (e) Overall survival after PSM. (f) Disease‐free survival after PSM.

The survival outcomes, including OS and DFS, were compared between the low CONUT‐GBRS and high CONUT‐GBRS groups using Kaplan–Meier survival analysis, as shown in Figure 1. Patients with low CONUT‐GBRS scores were inclined to share favorable OS (median OS: 30 months vs. 15 months, *p* < 0.001, Figure 1a) and DFS (median DFS: 28 months vs. 11 months, *p* < 0.001, Figure 1b). Furthermore, we investigated the applicability of the CONUT‐GBRS score to ICGB, and the results demonstrated that individuals with high CONUT‐GBRS had significantly bleak OS (*p* < 0.001, Figure 1c) and DFS (*p* < 0.001, Figure 1d).

PSM analysis was employed to address inherent selection bias and reduce variations in basic characteristics between the high and low CONUT‐GBRS groups. Given that the GBRS comprises four pathological parameters, the mentioned variables were excluded from the PSM. Following a 1:1 PSM process using variables including age, sex, CEA, CA19‐9, ICGB, postoperative complications, and node metastasis, the two groups were effectively balanced (Table 3). After PSM, the OS and DFS of individuals in the low CONUT‐GBRS group remained notably superior to those with high CONUT‐GBRS scores (Figure 1e,f).

**TABLE 3:** Baseline characteristics of GBC patients with different risk after propensity score matching.

Variables	High CONUT‐GBRS (*n* = 92)	Low CONUT‐GBRS (*n* = 92)	*p*
Age			
≤ 60	35 (38.0%)	34 (40.0%)	1.000
> 60	57 (62.0%)	58 (60.0%)	
Sex			
Male	36 (39.1%)	31 (33.7%)	0.540
Female	56 (60.9%)	61 (66.3%)	
BMI (Kg/m^2^)			
≤ 23	43 (46.7%)	44 (47.8%)	1.000
> 23	49 (53.3%)	48 (52.2%)	
CEA (ng/mL)			
≤ 5	64 (69.6%)	68 (73.9%)	0.624
> 5	28 (30.4%)	24 (26.1%)	
CA125 (U/mL)			
≤ 24	58 (63.0%)	62 (67.4%)	0.643
> 24	34 (36.0%)	30 (32.6%)	
CA19‐9 (U/mL)			
≤ 30	38 (41.3%)	43 (46.7%)	0.553
> 30	54 (58.7%)	49 (53.3%)	
Gallbladder stones			
Present	48 (52.3%)	55 (59.8%)	0.373
Absent	44 (47.7%)	37 (40.2%)	
Liver resection			
Yes	64 (69.6%)	64 (69.6%)	1.000
No	28 (30.4%)	28 (30.4%)	
Bile duct resection			
Yes	42 (45.7%)	35 (38.0%)	0.760
No	50 (54.3%)	47 (62.0%)	
ICGB			
Yes	26 (28.3%)	28 (30.4%)	0.610
No	66 (71.7%)	64 (69.6%)	
Postoperative complication			
Present	30 (27.0%)	28 (25.2%)	0.871
Absent	81 (73.0%)	83 (74.8%)	
Pathology			
Adenocarcinoma	79 (85.9%)	79 (85.9%)	1.000
Others	13 (14.1%)	13 (14.1%)	
Differentiation			
Poor	56 (58.6%)	37 (36.0%)	0.008
Moderate/well	36 (41.4%)	55 (64.0%)	
Perineural invasion			
Present	27 (29.3%)	17 (18.5%)	0.119
Absent	65 (70.1%)	75 (71.5%)	
Lymphovascular invasion			
Present	18 (19.6%)	16 (17.4%)	0.850
Absent	74 (70.4%)	76 (82.6%)	
Node metastasis			
Present	41 (44.6%)	28 (30.4%)	0.067
Absent	51 (55.4%)	64 (69.6%)	
pT (8th AJCC)			
T1/T2	62 (67.4%)	76 (82.6%)	0.012
T3	32 (32.6%)	16 (17.4%)	

Abbreviations: AJCC, American Joint Committee on Cancer; BMI, body mass index; CA125, carbohydrate antigen 125; CA19‐9, carbohydrate antigen 19‐9; CEA, carcinoembryonic antigen; ICGB, incidental gallbladder carcinoma.

**FIGURE 2:**
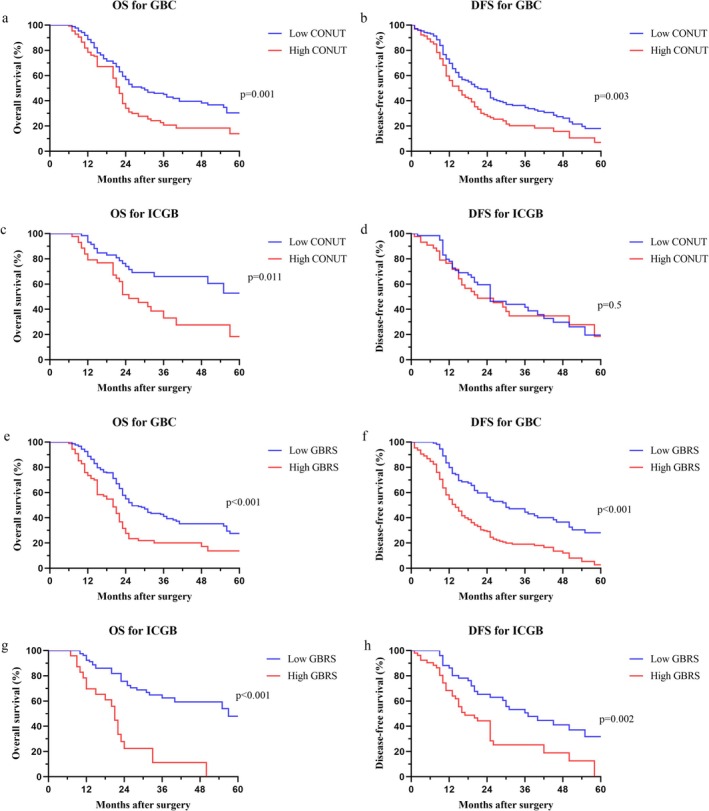
Comparison of survival outcomes of GBC patients stratified based on the CONUT score and GBRS. Overall survival (a) and disease‐free survival (b) of GBC stratified based on the CONUT score; overall survival (c) and disease‐free survival (d) of ICGB stratified based on the CONUT score; overall survival (e) and disease‐free survival (f) of GBC stratified based on the GBRS; overall survival (g) and disease‐free survival (h) of ICGB stratified based on the GBRS.

We also investigated the associations between CONUT, GBRS, and survival outcomes. Our results of Kaplan–Meier survival analyses suggested that GBC patients with high CONUT or GBRS shared dismal OS and DFS (Figure 2a,b,e,f). In addition, we further identified the prognostic importance of CONUT and GBRS in ICGB, and the results indicated that high CONUT was only significantly associated with OS, while high GBRS was notably correlated with both OS and DFS (Figure 2c,d,g,h).

### 3.5 Risk Factors Associated With Survival of GBC

**FIGURE 3:**
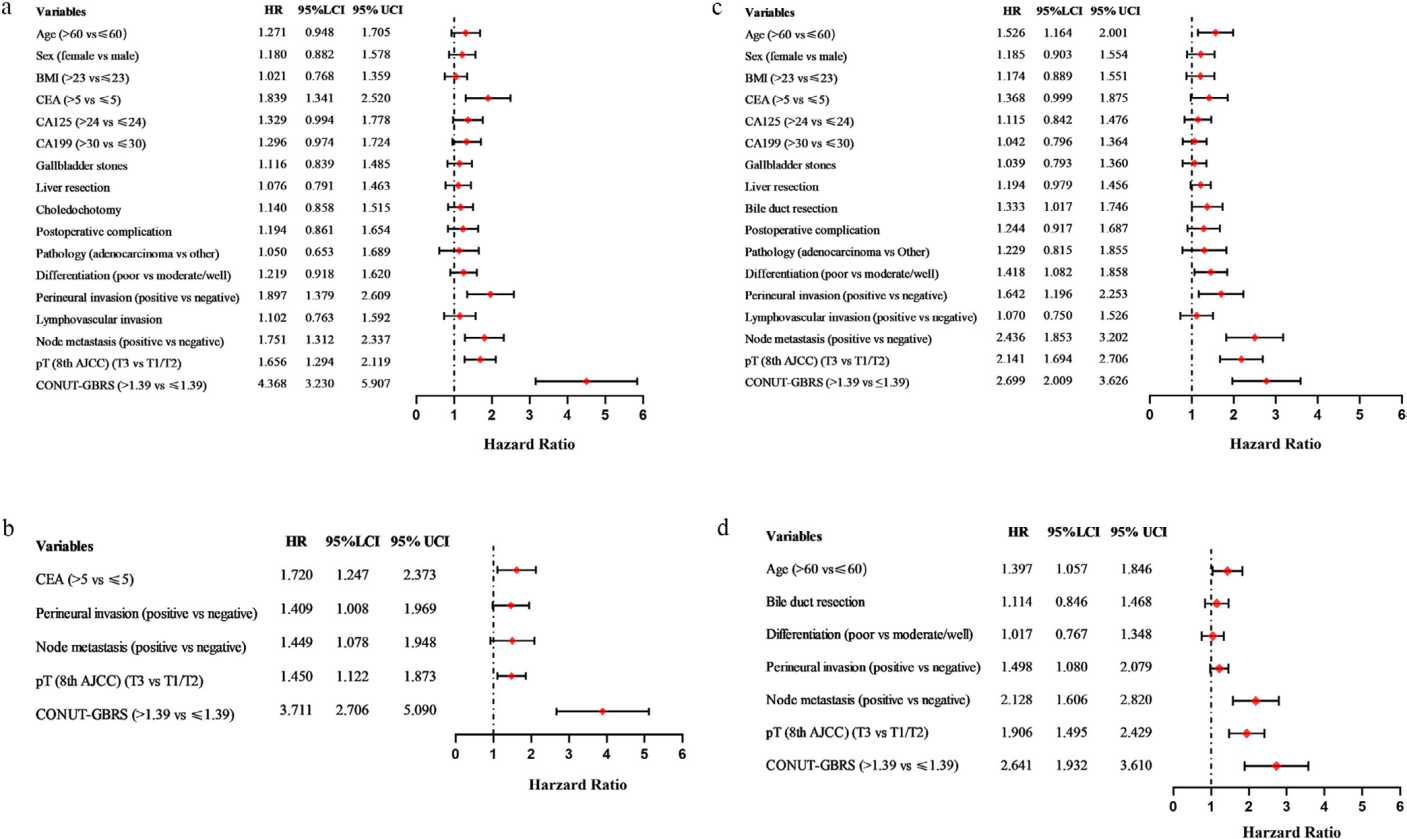
Cox regression analysis for overall survival and disease‐free survival. (a) Univariate analysis for overall survival. (b) Multivariate analysis for overall survival. (c) Univariate analysis for disease‐free survival. (d) Multivariate analysis for disease‐free survival.

The univariate analysis indicated that serum level of CEA > 5 ng/mL, presence of perineural invasion, presence of node metastasis, advanced T‐stage, and high CONUT‐GBRS were associated with poor OS (Figure 3a). The multivariate analysis exhibited that serum level of CEA > 5 ng/mL (HR: 1.720, 95% CI 1.247–2.373, *p* = 0.001), perineural invasion (HR: 1.409, 95% CI 1.008–2.969, *p* = 0.018), presence of node metastasis (HR: 1.449, 95% CI 1.078–1.948, *p* = 0.014), advanced AJCC T‐stage (HR: 1.450, 95% CI 1.122–1.873, *p* = 0.005) and CONUT‐GBRS > 1.39 (HR: 3.711, 95% CI 2.706–5.090, *p* < 0.001) were independent risk factors for OS in GBC patients (Figure 3b). Similarly, we found that age > 60 years, poor differentiation, presence of perineural invasion, presence of node metastasis, advanced AJCC T‐stage, and high CONUT‐GBRS were correlated with dismal DFS (Figure 3c). Further multivariate analysis unveiled that age > 60 years (HR: 1.397, 95% CI 1.057–1.846, *p* = 0.044), perineural invasion (HR: 1.498, 95% CI 1.080–2.079, *p* = 0.008), presence of node metastasis (HR: 2.128, 95% CI 1.606–2.820, *p* < 0.001), advanced AJCC T‐stage (HR: 1.906, 95% CI 1.495–2.429, *p* < 0.001) and CONUT‐GBRS > 1.39 (HR: 2.641, 95% CI 1.932–3.610, *p* < 0.001) were independently related to poor DFS (Figure 3d).

## 4 Discussion

Surgical resection is currently the only possible cure for GBC; however, considering that only a small percentage of patients have the opportunity for surgery because of the highly aggressive nature and tumor heterogeneity, despite undergoing radical resection, there are unfavorable rates of postoperative recurrence and long‐term survival. Numerous efforts have been undertaken to ascertain prognostic factors for GBC. Presently, the AJCC TNM staging system stands out as the most commonly employed prognostic tool, exhibiting substantial prognostic significance across various cancers. However, studies have reported that survival varies widely among patients in the same TNM stage [18, 19], indicating that the predictive capability of the AJCC TNM staging system might be insufficient for personalized survival assessments. Potential reasons for this could be that TNM staging fails to consider tumor heterogeneity, neglects the biology of highly aggressive tumors, and overlooks individual patient factors. Therefore, based on the patient's own state and tumor pathological features, we developed a novel prognostic model for resected GBC patients to assist in tailoring individualized treatment decisions.

This prognostic model included the CONUT score and GBRS. The CONUT score is derived from serum albumin and cholesterol levels, along with peripheral blood lymphocyte counts, providing an assessment of both the patient's nutritional status and systemic inflammatory condition. The prognostic importance of the CONUT score has been confirmed in diverse types of cancers. The nutritional and inflammatory states have been identified as closely linked to tumor progression and treatment tolerance [20]. Serum albumin, a crucial nutritional indicator, is widely acknowledged as a significant prognostic factor for numerous cancers [21, 22, 23]. Hypoalbuminemia is indicative of a weakened immune system, indicating that serum albumin is associated with the strength of the host immune system [24]. Lymphocytes are pivotal in cell‐mediated antitumor immunity and immune surveillance. Insufficient lymphocyte levels result in inadequate immune responses within the tumor microenvironment [25], contributing to a poorer prognosis in cancer patients [21]. Tumor tissues elevate low‐density lipoprotein (LDL) uptake, causing a reduction in serum total cholesterol concentration [26], which is linked to an unfavorable prognosis in cancer patients [27]. In this study, the CONUT score emerged as an independent prognostic marker, revealing an inverse relationship between the CONUT score and both OS and DFS of GBC patients. Therefore, we hypothesized that offering appropriate nutritional support to GBC patients with high preoperative CONUT scores might contribute to improving their prognosis.

The GBRS was initially designed to forecast the occurrence of local residual and distant diseases during reoperation for ICGB. An earlier study proposed that with an increase in the GBRS score, there was a corresponding rise in the incidence of local residual and distant diseases in patients with ICGB, accompanied by a decrease in OS [10]. Additionally, Mochizuki et al. demonstrated that the applicability of the GBRS extends beyond ICGB and can be utilized to predict the OS of all patients with GBC [5]. GBRS was computed based on T staging, tumor differentiation, and the presence of lymphovascular invasion and perineural invasion. The survival of tumor patients is widely recognized to be closely associated with T staging. It was reported that the 5‐year survival rates in GBC patients could reach 85.9% for stage T1, 56.1% for stage T2, but decreased significantly to 19.2% and 14.1% for stages T3 and T4, respectively [28]. Beyond T staging, tumor differentiation, lymphovascular invasion, and perineural invasion are additional pathological factors linked to the prognosis of GBC patients. Individuals with GBC exhibiting poor differentiation experienced inferior survival outcomes, and tumor differentiation has been identified as an independent risk factor for prognosis [29]. Lymphovascular invasion has been demonstrated to contribute to unfavorable prognoses in prostate cancer [30], colorectal cancer [31] and breast cancer [32]. There is a robust association between lymphovascular invasion and local lymph node metastasis as well as liver invasion in GBC. Furthermore, it is correlated with a reduction in OS and DFS [33, 34]. Our prior investigation affirmed the prognostic relevance of perineural invasion in individuals with GBC [8], aligning with findings from other research studies [35, 36]. Recent reviews have illuminated the connection between nerves and tumors, highlighting how nerves can influence tumorigenesis and progression, while tumors, in turn, can induce neurological remodeling and dysfunction [37, 38]. Perineural invasion arises from an existing network of nerve fibers, and cancer exploits this distribution network for metastasis. GBC cells were reported to disseminate to the tissues around the extrahepatic bile duct via perineural and lymphatic pathways [35].

Consistent with previous findings, our investigation revealed that both CONUT and GBRS scores independently served as prognostic factors for both OS and DFS in patients with GBC. In this research, a novel risk stratification system (CONUT‐GBRS) was devised for the comprehensive evaluation of GBC patients. The CONUT‐GBRS integrates both CONUT and GBRS, evaluating not only the aggressive characteristics of the tumor but also the patient's nutritional and immune status. Our findings suggest that GBC patients with elevated CONUT‐GBRS exhibited significantly inferior OS and DFS. Additionally, beyond the tumor aggressiveness features incorporated in GBRS, patients with high CONUT‐GBRS displayed unfavorable clinical characteristics, such as elevated serum levels of CEA and CA19‐9. Considering that variations in clinical characteristics can influence survival outcomes, we conducted a PSM analysis. Even after this analysis, significant survival disparities persisted between patients with high and low risk scores. Both univariate and multivariate Cox analyses established that CONUT‐GBRS stood as an independent prognostic factor for GBC patients. Furthermore, given that the GBRS was initially designed for ICGB patients, we evaluated the prognostic relevance of CONUT‐GBRS specifically in ICGB patients. The results showed that CONUT‐GBRS effectively stratified risk among ICGB patients, with those possessing low CONUT‐GBRS experiencing significantly extended OS and DFS. Therefore, we concluded that CONUT‐GBRS can serve as a reliable prognostic prediction tool for GBC patients, applicable to both ICGB and GBC cases. Therefore, more aggressive postoperative therapeutic strategies may be considered for GBC patients with high CONUT‐GBRS scores, including potentially relaxing the criteria for postoperative adjuvant therapy for these patients and ensuring closer follow‐up observation to identify the recurring patients at an early stage, thereby improving the prognosis of GBC.

Previous studies indicated that patients with advanced GBC often suffer from impaired nutritional and immune status due to obstructive jaundice and bile duct inflammation [39, 40]. A recent study reported that CONUT had an independent effect on the prognosis of patients with advanced bile tract cancer (BTC) treated with chemotherapy and/or immunotherapy [41]. In our study, only a small number of GBC patients with T3 were included, and we performed a subgroup analysis to investigate the applicability of CONUT‐GBRS in T2 and T3 GBC. Our results showed that patients with low CONUT‐GBRS scores still had better OS and DFS than those with high CONUT‐GBRS scores (Figure S1), indicating the good applicability of CONUT‐GBRS for T2 and T3 GBC. However, the role of our CONUT‐GBRS score remained unclear in T4 and unresectable GBC patients. On the one hand, there were no T4 GBC patients included in this study. For most GBC patients with T4 disease, radical surgical resection is much more extensive and is generally not recommended. Only selected patients, such as younger individuals with good physical status and lower perioperative risk, may be considered candidates for radical surgery [2, 3]. On the other hand, the parameters constituting the GBRS must be determined through postoperative pathological examination, making it challenging to obtain this data for unresectable patients. Therefore, additional studies are necessary to investigate the clinical value of CONUT‐GBRS in patients with T4 and unresectable GBC.

Current guidelines for surgical indications for secondary radical treatment of ICGB are based exclusively on AJCC T‐stage, recommending secondary radical resection for ICGB patients with T1b, T2, and T3 tumors [42]. However, several studies observed different survival outcomes in patients with the same AJCC T‐stage [6, 43], which suggested that the significance of AJCC T‐stage alone remained somewhat contentious. Ethun et al. demonstrated that the GBRS could be used as an indicator for secondary radical treatment in ICGB [10]. They suggested that surveillance might be better than re‐resection for T1b ICGB with low GBRS scores. Re‐resection may be appropriate in T1b patients with high GBRS scores and T2/T3 patients with low GBRS scores. However, for T2/T3 patients with high GBRS scores, secondary radical resection may not be the treatment of choice. Given that the CONUT‐GBRS score was developed based on the GBRS tool, we speculate that for patients with high CONUT‐GBRS scores, the decision regarding whether re‐resection is necessary should be considered alongside their T‐staging. Compared to GBRS, we believe that the CONUT‐GBRS might offer certain advantages as it integrates the patient's preoperative nutritional and immune status and pathological features, enabling a more comprehensive evaluation of both the patient's general condition and tumor aggressiveness features. However, the potential utility of CONUT‐GBRS as a marker for radical secondary resection of ICGB warrants validation through further investigations.

Our study holds significant clinical implications. Typically, researchers investigating GBC tend to concentrate on either tumor biology or the general condition of patients. However, our study assessed both patient general health and tumor pathology characteristics, revealing that the combined clinical prognostic value surpassed that of a single prognostic factor. This finding could potentially open new avenues for clinicians and oncologists. Nonetheless, our study comes with certain limitations. It was a retrospective study conducted at a single center, and inherent biases related to this study design are inevitable. The inclusion of patients exclusively from one provider and the exclusion of those with incomplete data might introduce some selection bias. These biases could potentially diminish the robustness of our findings. Furthermore, variations in the surgical approach, particularly in the extent of surgical resection, were noted among patients at different stages of GBC. Therefore, it is imperative to conduct more prospective or multicenter high‐quality studies with larger patient populations to validate the robustness of our findings.

## 5 Conclusion

In conclusion, this study affirms the prognostic significance of the CONUT‐GBRS in individuals undergoing radical surgery for GBC (encompassing ICGB). Notably, a high CONUT‐GBRS emerges as an independent risk factor associated with an unfavorable prognosis in GBC patients. The CONUT‐GBRS serves as an advantageous, straightforward, and cost‐effective prognostic tool for GBC, offering valuable prognostic insights and guiding the tailoring of individualized treatment strategies to improve patient outcomes. These findings require validation through subsequent prospective studies conducted across multiple centers and external verification.

## Author Contributions

**Si‐qi Yang:** investigation (lead), methodology (lead), writing – original draft (lead). **Rui‐qi Zou:** data curation (equal). **Yu‐shi Dai:** data curation (equal). **Jun‐ke Wang:** data curation (equal). **Wen‐jie Ma:** data curation (equal). **Hai‐jie Hu:** conceptualization (equal), writing – review and editing (equal). **Fu‐yu Li:** conceptualization (equal), funding acquisition (lead), writing – review and editing (equal).

## Ethics Statement

This study was approved by the Medical Ethics Committee of the West China Hospital of Sichuan University (20231394).

## Conflicts of Interest

The authors declare no conflicts of interest.

## Supporting information


**Appendix S1:** cam471203‐sup‐0001‐AppendixS1.docx.

## Data Availability

All data generated or analyzed during this study is included in the published article.
