# Supplementary material for: Individualized Prognostic Insights: CONUT‐GBRS for Survival Prediction in Gallbladder Cancer
Source: Cancer Med. 2025 Sep 12;14(18):e71203. doi: 10.1002/cam4.71203 (PMC12426482; doi:10.1002/cam4.71203)
Supplement: Supplementary file 1 — Appendix S1: cam471203‐sup‐0001‐AppendixS1.docx. [file CAM4-14-e71203-s001.docx]

**Table S1**. The controlling nutritional status (CONUT) scoring system

| **Parameters** | **Normal** | **Mild** | **Moderate** | **Severe** |
| --- | --- | --- | --- | --- |
| Serum albumin (g/dL) | ≥3.5 | 3.0-3.4 | 2.5-2.9 | < 2.50 |
| (Score) | 0 | 2 | 4 | 6 |
| Total lymphocyte (count/mm^3^) | ≥1600 | 1200-1599 | 800-1199 | < 800 |
| (Score) | 0 | 1 | 2 | 3 |
| Total cholesterol (mg/dl) | ≥180 | 140-179 | 100-139 | < 100 |
| (Score) | 0 | 1 | 2 | 3 |
| Total score | 0-1 | 2-4 | 5-8 | 9-12 |
| Dysnutritional states | Normal | Mild | Moderate | Severe |

**Table S2.** The gallbladder cancer predictive risk score (GBRS).

| **Parameters** | **Score** |
| --- | --- |
| **T stage** |  |
| Tis/T1a | 0 |
| T1b | 1 |
| T2 | 2 |
| T3/T4 | 3 |
| **Differentiation** |  |
| Well | 1 |
| Moderate | 2 |
| Poor | 3 |
| **LVI** |  |
| Negative | 1 |
| Positive | 2 |
| **PNI** |  |
| Negative | 1 |
| Positive | 2 |


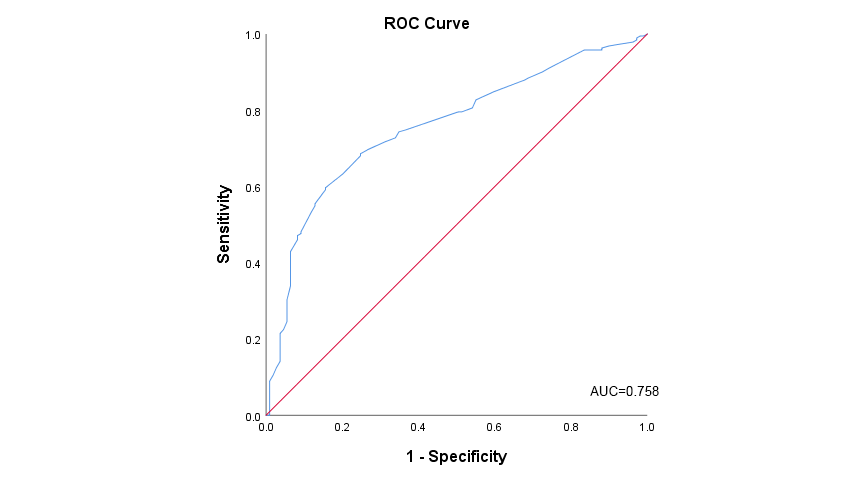


**Fig.S1:** Analysis of the ROC curve for predicting overall survival with CONUT-GBRS.


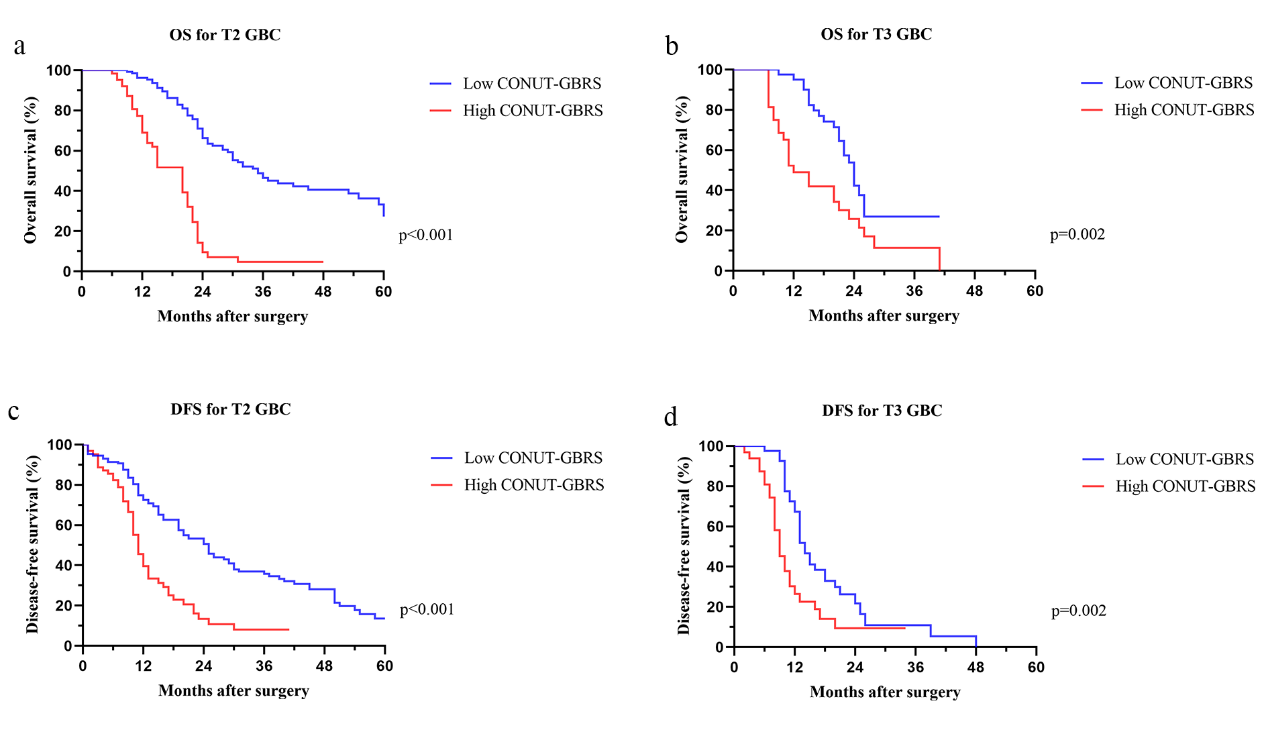


**Fig.S2**: Associations of CONUT-GBRS with the survival outcome of GBC patients (a): overall survival for T2 GBC; (b): overall survival for T3 GBC; (c): disease-free survival for T2 GBC; (d) disease-free survival for T3 GBC.
